# Hydrogen production and microbial kinetics of *Clostridium termitidis* in mono-culture and co-culture with *Clostridium beijerinckii* on cellulose

**DOI:** 10.1186/s13568-016-0256-2

**Published:** 2017-04-20

**Authors:** Maritza Gomez-Flores, George Nakhla, Hisham Hafez

**Affiliations:** 1 Department of Chemical and Biochemical Engineering, Faculty of Engineering, University of Western Ontario, London, ON N6A 5B9 Canada; 2 Department of Civil and Environmental Engineering, Faculty of Engineering, University of Western Ontario, London, ON N6A 5B9 Canada

**Keywords:** *Clostridium termitidis*, *Clostridium beijerinckii*, Co-culture, Hydrogen production, Cellulose, Microbial kinetics

## Abstract

Cellulose utilization by hydrogen producers remains an issue due to the low hydrogen yields reported and the pretreatment of cellulose prior to fermentation requires complex and expensive steps. *Clostridium termitidis* is able to breakdown cellulose into glucose and produce hydrogen. On the other hand, *Clostridium beijerinckii* is not able to degrade cellulose but is adept at hydrogen production from glucose; therefore, it was chosen to potentially enhance hydrogen production when co-cultured with *C. termitidis* on cellulose. In this study, batch fermentation tests were conducted to investigate the direct hydrogen production enhancement of mesophilic cellulolytic bacteria *C. termitidis* co-cultured with mesophilic hydrogen producer *C. beijerinckii* on cellulose at 2 g l^−1^ compared to *C. termitidis* mono-culture. Microbial kinetics parameters were determined by modeling in MATLAB. The achieved highest hydrogen yield was 1.92 mol hydrogen mol^−1^ hexose equivalent_added_ in the co-culture compared to 1.45 mol hydrogen mol^−1^ hexose equivalent_added_ in the mono-culture. The maximum hydrogen production rate of 26 ml d^−1^ was achieved in the co-culture. Co-culture exhibited an overall 32 % enhancement of hydrogen yield based on hexose equivalent added and 15 % more substrate utilization. The main metabolites were acetate, ethanol, lactate, and formate in the mono-culture, with also butyrate in the co-culture. Additionally, the hydrogen yield of *C. beijerinckii* only in glucose was 2.54 mol hydrogen mol^−1^ hexose equivalent. This study has proved the viability of co-culture of *C. termitidis* with *C. beijerinckii* for hydrogen production directly from a complex substrate like cellulose under mesophilic conditions.

**Electronic supplementary material:**

The online version of this article (doi:10.1186/s13568-016-0256-2) contains supplementary material, which is available to authorized users.

## Introduction

Hydrogen (H_2_) is considered a clean and renewable energy resource that does not contribute to the greenhouse effect (Lee et al. 2014). The main source of H_2_ production from fermentation is carbohydrates, among which, cellulose is widely available in agricultural wastes and industrial effluents such as pulp/paper and food industries (Lee et al. 2014). In comparison to the use of natural mixed consortia, pure cultures have achieved higher H_2_ yields (Masset et al. 2012). Artificial microbial co-cultures and consortia can perform complex functions (Masset et al. 2012), such as, simultaneous hexose and pentose consumption (Eiteman et al. 2008), maintaining anaerobic conditions for obligate H_2_ producers, improving the hydrolysis of complex sugars, allowing fermentation over a wider pH range (Elsharnouby et al. 2013), and could be more robust to changes in environmental conditions (Brenner et al. 2008). Although, thermophiles have shown higher H_2_ production yields than mesophiles in the literature (Kumar and Das 2000; Lu et al. 2007; Munro et al. 2009; Ngo et al. 2012), mesophilic H_2_ production is more economical and reliable than thermophilic and hyperthermophilic production. Four co-culture experiments for biohydrogen production from pure cellulose, two at mesophilic and two at thermophilic conditions (Geng et al. 2010; Liu et al. 2008; Wang et al. 2008, 2009) have been reported. All of these studies have shown enhancement of H_2_ production compared to mono-cultures, with the highest H_2_ yield of 1.8 mol hydrogen mol^−1^ hexose achieved by the co-culture of *Clostridium thermocellum* JN4 and *Thermoanaerobacterium thermosaccharolyticum* GD17 at 60 °C (Liu et al. 2008), potentially due to synergism between the two cultures.

*Clostridium termitidis* ATCC 51846 is an anaerobic, mesophilic, cellulolytic bacterium isolated from the gut of a termite (Hethener et al. 1992), with reported H_2_ yields of 1.99 mol hydrogen mol^−1^ hexose from glucose, 1.11 mol hydrogen mol^−1^ hexose equivalent from cellobiose (Gomez-Flores et al. 2015), and 0.62 mol hydrogen mol^−1^ hexose equivalent from cellulose (Ramachandran et al. 2008). On the other hand, *C. beijerinckii* is a mesophilic H_2_ producer which is not able to degrade cellulose but is adept at H_2_ production from glucose (Masset et al. 2012). *Clostridium beijerinckii* H_2_ yields from glucose have been reported to be 1.9 and 2.8 mol hydrogen mol^−1^ hexose_added or consumed_ (Lin et al. 2007; Masset et al. 2012), 2.5 mol hydrogen mol^−1^ hexose_consumed_ (Pan et al. 2008), and 2 mol hydrogen mol^−1^ hexose_added_ (Taguchi et al. 1992). These experiments differ from each other in the reactor size, medium and initial glucose concentration.

Additionally, reasonably accurate mathematical models able to predict biochemical phenomena as well as the determination of its parameters are essential since they provide the basis for design, control, optimization and scale-up of process systems (Huang and Wang 2010). Therefore, this study has two goals (1) evaluate the effect of co-culture of *C. termitidis* and *C. beijerinckii* on biohydrogen production and, (2) determine the microbial kinetics of *C. termitidis* in mono-culture and co-cultured with *C. beijerinckii* on cellulose.

## Materials and methods

### Microbial strain and media

The strains used were *C. termitidis* ATCC 51846 (American Type Culture Collection) and *C. beijerinckii* DSM 1820 (Deutsche Sammlung von Mikroorganismen und Zellkulturen). All chemicals for media and substrates were obtained from Sigma-Aldrich Canada Co. (Oakville, ON, Canada). Fresh cells of *C. termitidis* were maintained by successively transferring 10 % (v/v) of inoculum to ATCC 1191 medium containing 2 g l^−1^ of cellulose, whereas fresh cells of *C. beijerinckii* were maintained by successively transferring 10 % (v/v) of inoculum to ATCC 1191 medium containing 2 g l^−1^ of cellobiose. The ATCC 1191 medium was prepared according to Gomez-Flores et al. (2015).

### Experimental conditions

Batch fermentations were performed in media bottles (Wheaton, NJ, USA) with a working liquid volume of 500 and 210 ml of headspace. For the co-culture experiments, bottles containing 450 ml of ATCC 1191 medium and 1 g cellulose were tightly capped with screw caps with butyl septum, degassed by applying vacuum, sparged with high purity N_2_ gas, and autoclaved. Mono-culture bottles were inoculated with 10 % (v/v) of *C. termitidis* cultures, while co-culture bottles were inoculated with 10 % (v/v) of *C. termitidis* and *C. beijerinckii* cultures in a volumetric ratio of 1:1. All bottles were incubated at 37 °C in shakers (Max Q4000, Thermo Scientific, CA, USA). Three (3) ml liquid samples were taken at specific times for pH, metabolites, cellular protein content and cellulose analyses. Fermentations ran for 45 and 40 days for the mono-culture and co-culture, respectively. A total of 24 samples were taken for the mono-culture experiments whereas 21 samples were taken for the co-culture experiments. pH was initially set to 7.2 but was not controlled. Data shown are the averages of duplicate experiments. Additionally, fermentation on glucose 2 g l^−1^ by *C. beijerinckii* in the ATCC 1191 medium was performed in serum bottles (Wheaton, NJ, USA) with a working volume of 500 and 210 ml of headspace. Duplicate bottles were inoculated with 10 % (v/v) of fresh cultures. Bottles were incubated at 37 °C and 100 rpm for 48 h. Also, the initial pH was set to 7.2 but was not controlled.

### Analytical methods

Cell growth was monitored by measuring cellular protein content, samples (1 ml) were placed in microcentrifuge tubes (VWR^®^, Polypropylene) and centrifuged (Corning^®^ LSE™, NY, USA) at 10,000×*g* for 15 min. Supernatants were used for soluble product analysis by transferring to new microcentrifuge tubes. The pellets were re-suspended with 0.9 % (w/v) NaCl and centrifuged at the same aforementioned conditions. Supernatants were discarded, and 1 ml of 0.2 M NaOH was added to microcentrifuge tubes and vortexed to re-suspend the pellet. Microcentrifuge tubes were placed in a water bath at 100 °C for 10 min. After cooling, tubes were centrifuged and supernatants were collected for Bradford assay using bovine serum albumin (BSA) as standard, measured by a UV–visible spectrophotometer (Cary 50 Bio, Varian, Australia) at 595 nm. The cellulose pellet was quantified gravimetrically after being dried overnight at 100 °C (Liu et al. 2008). pH was measured using a B10P SympHony pH meter (VWR^®^). Ethanol, glucose, cellobiose, and lactic, formic, acetic, and butyric acids, were measured as follows: supernatants for metabolites analysis were filtered through 0.2 µm and measured using an HPLC (Dionex, Sunnyvale, CA, USA) consisting of a Dionex GP50 Gradient pump and a Dionex LC25 Chromatography oven equipped with an Aminex HPX-87H column (Bio-Rad) at 30 °C and 9 mM H_2_SO_4_ at 0.6 ml min^−1^ as mobile phase, connected to a Perkin Elmer 200 series refractive index detector (RID). Standard curves of metabolites, glucose and cellobiose were performed on ATCC 1191 medium. Cellular protein content was then converted to dry weight using the correlation dry weight (g l^−1^) = 0.0051 × protein (µg ml^−1^) (Gomez-Flores et al. 2015). For the estimation of the COD equivalents for the biomass dry weight, the empirical formula of the organic fraction of the biomass of C_5_H_7_O_2_N (Metcalf and Eddy 2003), and an organic fraction of 90 % of the cell dry weight (Pavlostathis et al. 1988), were assumed.

### Gas measurements

Gas volume was measured by releasing the gas pressure in the bottles using appropriately sized glass syringes in the range of 5 to 100 ml to equilibrate with the ambient pressure (Owen et al. 1979). H_2_ analysis was conducted by employing a gas chromatograph (Model 310, SRI Instruments, Torrance, CA, USA) equipped with a thermal conductivity detector (TCD) and a molecular sieve column (Mole sieve 5A, mesh 80/100, 1.83 m × 0.32 cm). The temperatures of the column and the TCD detector were 90 and 105 °C, respectively. Argon was used as the carrier gas at a flow rate of 30 ml/min.

### Modified Gompertz model

The following modified Gompertz model (Lay et al. 1999) was used to describe the H_2_ production.

1 $$ H\; = \;P\;exp\;\left\{ { - exp\left[ {\frac{{R_{max} \;e}}{P}\;\left( {\lambda \; - \;t} \right)\; + \;1} \right]} \right\} $$

where H is the cumulative H_2_ production (ml), P is the H_2_ production potential (ml), R_max_ is the maximum H_2_ production rate (ml d^−1^) and λ is the lag time (d).

### Kinetic equations and modeling

**Fig. 1:**
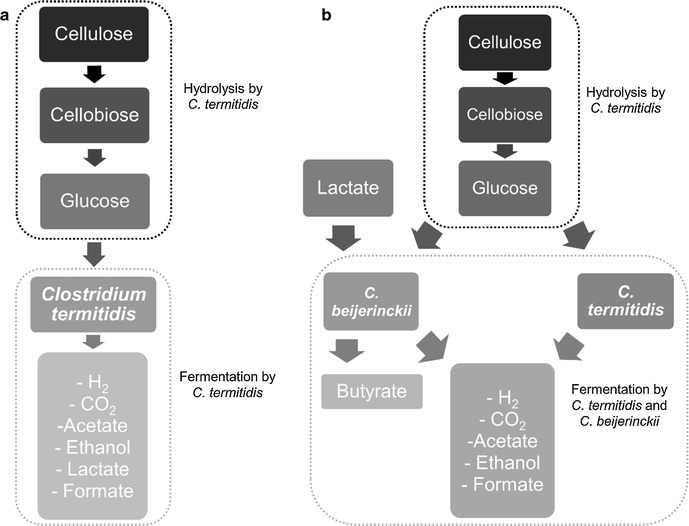
Schematic representation of the steps involved in cellulose fermentation in **a** mono-culture and **b** co-culture

As shown in Fig. 1, there are mainly 2 steps: hydrolysis of cellulose and fermentation of soluble sugars (glucose). In both cases, *C. termitidis’* putative cellulosome (Munir et al. 2014) is responsible for the cellulose hydrolysis. Fermentation of soluble sugars is performed by *C. termitidis* in mono-culture, whereas in co-culture both, *C. termitidis* and *C. beijerinckii* ferment the soluble sugars. The soluble products in mono-culture are acetate, ethanol, lactate and formate. In the co-culture, the lactate present in the *C. beijerinckii* growth media acted as substrate, and butyrate was an additional soluble product.

Among the various reactions involving glucose, only acetate and butyrate pathways involve H_2_ production according to Eqs. 2 and 3, respectively, while ethanol and lactate are involved in a zero-H_2_ balance (Guo et al. 2010)

2 $$ C_{6} H_{12} O_{6} \; + \;2H_{2} O\; \to \;2CH_{3} COOH\; + \;2CO_{2} \; + \;4H_{2} $$

3 $$ C_{6} H_{12} O_{6} \; \to \;CH_{3} CH_{2} CH_{2} COOH\; + \;2CO_{2} \; + \;2H_{2} . $$

Lactate utilization is represented by Eq. 4 (Thauer et al. 1977).

4 $$ CH_{3} CHOHCOOH\; + \;H_{2} O\; \to \;CH_{3} COOH\; + \;CO_{2} \; + \;2H_{2} . $$

Because cellulose was not completely biodegraded, the use of a non-biodegradable factor S_o_ (g COD l^−1^) was needed as presented in Eq. 5.

5 $$ S\; = \;\mathop \int \limits_{0}^{t} \frac{dS}{dt}\; + \;S_{o} $$

where S is cellulose concentration (g COD l^−1^) and S_o_ is the non-biodegradable cellulose concentration remaining at the end of the fermentation. Soluble sugars from cellulose hydrolysis (cellobiose and glucose) were not detected in any of the fermentations, implying that cellulose hydrolysis was the rate-limiting step. Nevertheless, cellulose is an insoluble substrate and Monod model cannot be used. Therefore, a modified Monod approach, incorporating particulate organic matter (POM) (Metcalf and Eddy 2003) was used (Eq. 6).

6 $$ {{\upmu }}\; = \;\frac{{{{\upmu }}_{{\varvec{max}}} \;\left( {\frac{{\varvec{PO}}}{\varvec{X}}} \right)}}{{\varvec{K}_{\varvec{X}} \; + \;\left( {\frac{{\varvec{PO}}}{\varvec{X}}} \right)}} $$

where µ_max_ (d^−1^) is the maximum specific growth rate, K_x_ is the half-velocity degradation coefficient (g COD PO g^−1^ COD biomass), PO is the particulate organic (cellulose) concentration (g COD l^−1^) and X is biomass concentration (g COD l^−1^) (Metcalf and Eddy 2003). The POM modeling approach considers the particulate substrate conversion rate as the rate-limiting process that is dependent on the particulate substrate and biomass concentrations. The particulate degradation concentration is expressed relative to the biomass because the particulate hydrolysis is related to the relative contact area between the non-soluble organic material and the biomass (Metcalf and Eddy 2003). All concentrations were expressed as g COD; for biomass the factor of 1.42 g COD g^−1^ biomass based on the empirical formula of C_5_H_7_O_2_N was used (Metcalf and Eddy 2003).

The two models are described as follows:

- *Mono-culture* (*C. termitidis* only). Biomass growth and PO consumption are described in Eqs. 7 and 8, respectively.
  7 $$ \frac{dX}{dt}\; = \;{{\upmu }}X\; = \;\frac{{{\upmu{ }}_{max} \;\left( {\frac{PO}{X}} \right)\;X}}{{\left[ {K_{X} \; + \;\left( {\frac{PO}{X}} \right)} \right]}} $$
  8 $$ \frac{dPO}{dt}\; = \; - \;\frac{{{{\upmu }}_{max} \;\left( {\frac{PO}{X}} \right)\;X}}{{Y_{{{X} / _{{PO}}}} \;\left[ {K_{X} \; + \;\left( {\frac{PO}{X}} \right)} \right]}} $$
  where Y_X/PO_ (g COD biomass g^−1^ COD PO) is the biomass yield (Shuler and Kargı 2002). Acetate, ethanol, lactate and formate production was modeled as described by Eq. 9.
  9 $$ \frac{dP}{dt}\; = \;\frac{{Y_{P} /_ {{PO}} }}{{Y_{{{{X}/_ {PO}}}} }}\; \frac{{{{\upmu }}_{max} \;\left( {\frac{PO}{X}} \right)\;X}}{{\left[ {K_{X} \; + \;\left( {\frac{PO}{X}} \right)} \right]}} $$
  where P and Y_P/PO_ are acetate, ethanol, lactate and formate concentrations (g COD l^−1^) and yields (g COD g^−1^ COD PO), respectively.
- *Co-culture* (*C. termitidis* and *C. beijerinckii*). No distinction in biomass measurement was done for each strain. Co-culture was modeled as a single strain with the addition of lactate as substrate and butyrate as product. Consequently, PO consumption is described in Eq. 8, biomass growth from cellulose and lactate is modeled by Eq. 10, and lactate consumption was considered a first order reaction (Eq. 11).
  10 $$ \frac{dX}{dt}\; = \;\frac{{{{\upmu }}_{max} \;\left( {\frac{PO}{X}} \right)\;X}}{{\left[ {K_{X} \; + \;\left( {\frac{PO}{X}} \right)} \right]}}\; + \;Y_{{X} {/ {L}}} K_{L} LX $$
  11 $$ \frac{dL}{dt} = - K_{L} LX $$
  where Y_X/L_ is the biomass yield from lactate (as g COD g^−1^ COD) and K_L_ is the lactate consumption constant (l g^−1^ COD biomass d^−1^). Based on Eq. 4, acetate is also produced from lactate. Thus acetate kinetics are modeled by Eq. 12.
  12 $$ \frac{dA}{dt}\; = \;\frac{{Y_{{{{A} {/ {{ {PO}}}} }}} }}{{Y_{X}{{{{/ _{{ {PO}}}} }}} }}\;\frac{{{{\upmu }}_{max} \;\left( {\frac{PO}{X}} \right)X}}{{\left[ {K_{X} \; + \;\left( {\frac{PO}{X}} \right)} \right]}}\; + Y_{A}/_{L} K_{L}LX$$
  where Y_A/L_ is the acetate yield from lactate (g COD g^−1^ COD).

Ethanol, formate and butyrate were described by Eq. 9, where P and Y_P/PO_ are also butyrate concentration (g COD l^−1^) and yield (g COD g^−1^ COD PO).

Microbial kinetics were estimated from the growth phase only, ignoring the lag phase. Kinetic parameters were estimated using MATLAB^®^ R2014a. The solver function used for numerical integration of the ordinary differential equations i.e. Ode45, implemented fourth/fifth order Runge–Kutta methods. Initial guesses were manually adjusted to obtain a good fit to the data, and average percentage errors (APE) and root mean square errors (RMSE) were calculated. The complete nomenclature is shown in Table 1.

**Table 1:** Abbreviations

Parameter	Meaning and units
K_L_	Lactate consumption constant (l g^−1^ COD biomass d^−1^)
K_m_	Substrate utilization rate (g COD PO g^−1^ COD biomass d^−1^)
K_x_	Half-velocity degradation coefficient (g COD PO g^−1^ COD biomass)
µ_max_	Maximum specific growth rate (d^−1^)
S_o_	Non-biodegradable factor (g COD l^−1^)
Y_A/L_	Acetate yield from lactate (g COD g^−1^ COD lactate)
Y_A/PO_	Acetate yield from particulate organic (g COD g^−1^ COD PO)
Y_B/PO_	Butyrate yield from particulate organic (g COD g^−1^ COD PO)
Y_E/PO_	Ethanol yield from particulate organic (g COD g^−1^ COD PO)
Y_F/PO_	Formate from particulate organic (g COD g^−1^ COD PO)
Y_L/PO_	Lactate yield from particulate organic (g COD g^−1^ COD PO)
Y_X/L_	Biomass yield from lactate (g COD g^−1^ COD lactate)
Y_X/PO_	Biomass yield from particulate organic (g COD biomass g^−1^ COD PO)

## Results

### *C. beijerinckii* on glucose experiment

*Clostridium beijerinckii* degraded glucose in 46 h with an initial lag phase of 22 h and had a yield of 2.54 mol hydrogen mol^−1^ glucose (Additional file 1: Figure S1a). pH dropped from 7.1 to 6.2. With a 28 % higher H_2_ yield over *C. termitidis* for the same substrate (Gomez-Flores et al. 2015) and under the same operating conditions, with the exception of using 500 ml of working volume instead of 400 ml, *C. beijerinckii* was chosen to potentially enhance H_2_ production when co-cultured with *C. termitidis* on cellulose by serving as a high H_2_ producer from glucose formed from cellulose hydrolysis by *C. termitidis*.

At the same time, a correlation between dry weight and cellular protein content was developed for *C. beijerinckii* in a similar way to the correlation for *C. termitidis* (Gomez-Flores et al. 2015). A 20 % cellular protein content was obtained, in close agreement with the 19 % obtained for *C. termitidis* in the aforementioned study (Additional file 1: Figure S1b).

### Hydrogen production from cellulose

**Fig. 2:**
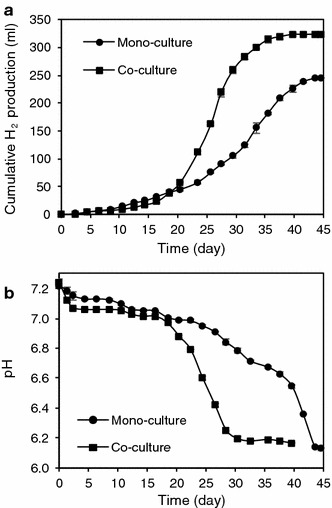
*C. termitidis* mono-cultured in 2 g l^−1^ cellulose and co-cultured with *C. beijerinckii* 2 g l^−1^ cellulose. **a** Cumulative H_2_ production profiles. **b** pH profiles. Data points are the averages of duplicates, *lines above* and *below* represent the actual duplicates

The H_2_ production profiles in Fig. 2a clearly depict the enhancement in H_2_ production from co-culture over mono-culture. H_2_ production showed long lag phases of up to 17 days. The results of the modified Gompertz model are shown in Table 2. The overall H_2_ production for the co-culture compared with the mono-culture increased by 30 % to 326 ml. Moreover, the H_2_ production rate in the co-culture of 26 ml d^−1^ was double the 12 ml d^−1^ observed in the mono-culture.

**Table 2:** H_2_ yields and Gompertz parameters of *C. termitidis* mono-cultured and co-cultured with *C. beijerinckii* on 2 g l^−1^ cellulose

	Cellulose consumed (%)	H_2_ yields		Gompertz parameters			
		mol H_2_ mol^−1^ hexose eq._added_	mol H_2_ mol^−1^ hexose eq_consumed_	P_max_^a^ (ml)	R_m_^b^ (ml d^−1^)	λ^c^ (d)	R^2^
Mono	81	1.45	1.8	250	12	19	0.98
Co	93	1.92	2.05	326	26	19	0.99

^a^H_2_ production potential

^b^Maximum H_2_ production rate

^c^Lag phase

Figure 2b shows the pH profiles. During the lag phases, all cultures exhibited a marginal decrease in pH from 7.2 to around 7. Concurrent with the H_2_ production, the pH dropped to around 6.1. As the optimum pH range for *C. termitidis* growth has been reported to be >5 to <8.2 (Hethener et al. 1992), the pH changes observed in mono-culture fermentations were assumed not to impact the microbial kinetics. For *C. beijerinckii* DSM 1820 growth, the pH range reported is from 5.2 to 7.3, with the former reported as inhibitory (Masset et al. 2012). As the observed pH changes in the co-culture fermentation were within the growth range reported for both strains, pH changes were assumed not to affect the microbial kinetics.

Cellulose was not completely consumed in neither case but co-culture enhanced the extent of cellulose utilization by 15 % to about 93 % (Table 2).

Table 2 also shows the H_2_ yields based on hexose equivalent added and consumed. The H_2_ yield of 1.92 mol hydrogen mol^−1^ hexose equivalent_added_ obtained in the co-culture was 32 % greater than the H_2_ yield obtained by the mono-culture of 1.45 mol hydrogen mol^−1^ hexose equivalent_added_. Also, the H_2_ yield of 2.05 mol hydrogen mol^−1^ hexose equivalent_consumed_ in the co-culture was 14 % greater than the H_2_ yield obtained by the mono-culture of 1.8 mol hydrogen mol^−1^ hexose equivalent_consumed_.

### Microbial products and kinetics

**Fig. 3:**
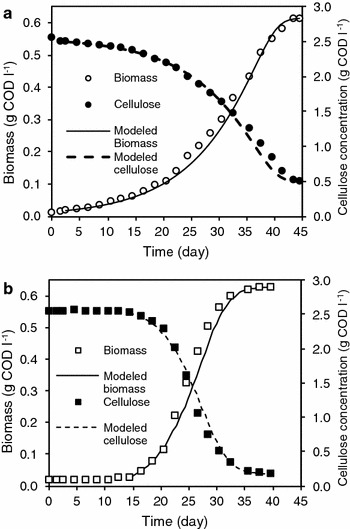
Experimental and modeled growth kinetics. **a** Mono-culture. **b** Co-culture

The experimental and modeled biomass and cellulose profiles are illustrated in Fig. 3, which emphatically demonstrates that the co-culture was able to utilize more cellulose than mono-culture and the ultimate biomass growth was similar in all cases.

**Fig. 4:**
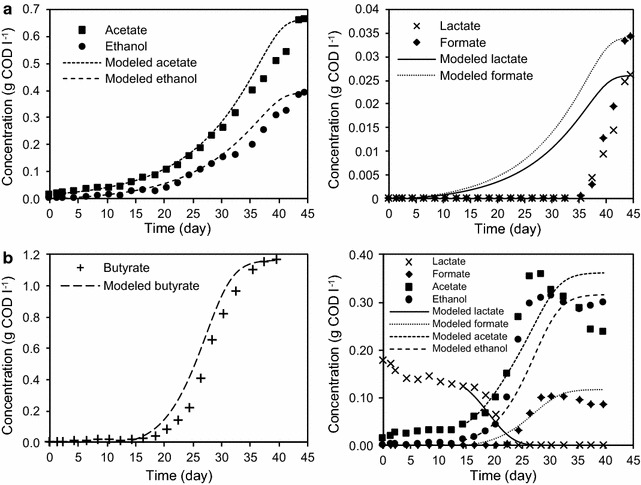
Experimental and modeled profile of metabolites in **a** mono-culture and **b** co-culture

Figure 4 shows the experimental and modeled metabolites profiles. Neither glucose nor cellobiose from cellulose hydrolysis were detected in any of the fermentations, implying that cellulose hydrolysis was the rate limiting factor. *Clostridium termitidis* metabolites on cellulose were acetate, ethanol, lactate, and formate, in agreement with Ramachandran et al. (2008). In mono-culture experiments, acetate and ethanol were produced during biomass growth, while, formate and lactate exhibited lag phases and were not detected until day 38. H_2_ production peaked around day 44 for the mono-culture experiment, concurrent with all metabolites peak.

*Clostridium beijerinckii* DSM 1820 soluble products from glucose have been reported by Masset et al. (2012) to be butyrate, acetate, formate, lactate, in addition to butanol, acetone and isopropanol by Chen and Hiu (1986), although, other strains of *C. beijerinckii* (i.e. L9 and Fanp3) have been demonstrated to produce ethanol from glucose (Lin et al. 2007; Pan et al. 2008). In the co-culture experiment acetate and butyrate were produced as lactate was consumed. It is noteworthy that only butyrate production peaked on day 40, concurrent with the H_2_ peak in the co-culture.

Mathematical models that accurately predict biochemical phenomena provide the basis for design, control, optimization and scale-up of process systems (Huang and Wang 2010). Kinetic parameters of the mathematical model are shown in Table 3. The co-culture exhibited the highest µ_max_ (0.2 d^−1^), thus rationalizing the end of the fermentation test before the mono-culture. In this regard, the impact of the synergy in microbial kinetics was notorious, with µ_max_ in co-culture of 0.2 d^−1^ double the 0.1 d^−1^ observed in mono-culture. It is noteworthy that the maximum specific growth rates achieved on glucose and cellobiose by *C. termitidis* of 0.22 and 0.24 h^−1^, respectively (Gomez-Flores et al. 2015), are more than 50 times greater than those achieved by the same strain on cellulose. The half-saturation constant, K_x_, varied between 0.42 and 1.1 g COD cellulose g^−1^ COD biomass. PO/X values (Additional file 1: Figure S2) are significantly greater than the Kx values, i.e. the growth rate throughout the experiments equals µ_max_. The recommended value for the hydrolysis rate of carbohydrates in the anaerobic digestion model (ADM1) (Batstone et al. 2002) is 0.25 d^−1^ at mesophilic conditions which is comparable to the growth rates obtained in the present study, clearly emphasizing that the biodegradation of cellulose is hydrolysis-limited.

**Table 3:** Kinetic parameters obtained in MATLAB of *C. termitidis* mono-cultured and co-cultured with *C. beijerinckii* on 2 g l^−1^ cellulose

	Mono-culture	Co-culture
$$ S_{o}$$ ^a^ $$ ({\text{g COD}} \,{\text{l}}^{ - 1} ) $$	0.49	0.17
$\upmu_{max} ({\text{d}}^{ - 1} )$	0.10	0.20
$$ Y_{{x}{/ { L}}}$$ ^b^	NA	0.25
$$ Y_{{x/PO}}$$ ^c^	0.30	0.25
*K*_*m*_^d^	0.33	0.80
$Y_{L/PO}$ ^e^	0.013	NA
$Y_{F/PO}$ ^f^	0.017	0.05
$Y_{A/PO}$ ^g^	0.32	0.11
$Y_{E/PO}$ ^h^	0.194	0.13
$Y_{B/PO}$ ^i^	0	0.49
$Y_{A/L}$ ^j^	NA	0.49
*K*_*L*_	NA	2.5
*K*_*x*_^k^	0.42	1.1

*NA* Not applicable

^a^Non-biodegradable factor

^b^Biomass yield from lactate (g COD g^−1^ COD lactate)

^c^Biomass yield (g COD g^−1^ COD PO)

^d^g COD PO g^−1^ COD biomass d^−1^

^e^Lactate yield (g COD g^−1^ COD PO)

^f^Formate yield (g COD g^−1^ COD PO)

^g^Acetate yield (g COD g^−1^ COD PO)

^h^Ethanol yield (g COD g^−1^ COD PO)

^i^Butyrate yield (g COD g^−1^ COD PO)

^j^Acetate yield from lactate (g COD g^−1^ COD lactate)

^k^g COD PO g^−1^ COD biomass

Co-culture experiment reflected a slightly lower biomass yield than monoculture (0.25 vs 0.3 g COD g^−1^ COD cellulose). Y_X/L_ (biomass yield from lactate) was assumed to be the same as Y_X/PO_ (biomass yield from cellulose) and Y_A/L_ (acetate yield from lactate) was calculated as follows:

14 $$Y_{A/L}\,=\,f_{A/L}(1-Y_{X/L})$$

where f_A/L_ is the stoichiometric relationship based on Eq. 4 of 1 mol acetate per mol lactate, calculated in g COD as 0.66. Y_A/L_ was calculated to be 0.49 g COD acetate g^−1^ COD lactate and the theoretical H_2_ production from lactate was also calculated based on Eq. 4 and subtracted from the measured H_2_ produced. The modified H_2_ yields from cellulose in the co-culture experiment were 1.72 mol hydrogen mol^−1^ hexose equivalent_added_ and 1.84 mol hydrogen mol^−1^ hexose equivalent_consumed_, approximately 19 % higher than the mono-culture based on hexose added. Nevertheless, the calculated H_2_ from lactate may be overestimated since it is theoretical.

The average percentage errors (APE) and RMSE calculated for the modeled biomass, substrate and metabolites are the in Additional file 1: Table S1. Biomass and cellulose exhibited the lowest average percentage errors, within the range of 4–8 %, followed by PO/X with the highest value of 11 % in co-culture. For both lactate and formate in mono-culture, the model significantly under estimated the lag phase, as evident from Fig. 4a. Accordingly, the APE excluding the lag phase for lactate and formate were 12 and 11 % and including lag phases was 81 % in both cases.

## Discussion

### Hydrogen production

COD balances calculated by summation of metabolites, H_2_, cellulose and cells as g COD l^−1^ at the beginning and end of fermentations are presented in Table 4. The COD balances closed within 3–8 % of the initial, thus confirming the reliability of the data. Theoretical H_2_ production from acetate and butyrate shown in Table 4 was calculated based on 848 ml hydrogen g^−1^ acetate and 578 ml hydrogen g^−1^ butyrate (Eqs. 2, 3). The theoretical values were consistent with the H_2_ measured during the experiment with an average percent difference of 1 % of the theoretical H_2_. *C. beijerinckii* DSM 1820 produced a H_2_ yield of 2.54 mol hydrogen mol^−1^ glucose, added or consumed, in line with the 1.9 and 2.8 mol hydrogen mol^−1^ hexose_added or consumed_ (Lin et al. 2007; Masset et al. 2012), 2.5 mol hydrogen mol^−1^ hexose_consumed_ (Pan et al. 2008), and 2 mol hydrogen mol^−1^ hexose_added_ (Taguchi et al. 1992). On the other hand, while the highest reported mesophilic H_2_ yield by co-culture on cellulose is 1.31 mol H_2_ mol^−1^ hexose with *Clostridium acetobutylicum* X9 and *Ethanoigenens harbinense* B49 (Wang et al. 2008), and the highest thermophilic H_2_ yield is 1.8 mol H_2_ mol^−1^ hexose with *C. thermocellum* JN4 and *T. thermosaccharolyticum* GD17 (Liu et al. 2008), the results from this study (Table 2) reveal a significantly improved H_2_ yield in the co-culture of *C. termitidis* and *C. beijerinckii* compared to the literature. The achievement of a yield of 1.92 mol hydrogen mol^−1^ hexose using two mesophilic cultures represents about 50 % improvement of the literature at similar conditions. Although the aforementioned yield is only 7 % higher than the maximum thermophilic yield, the balance of thermal energy input and output based on hydrogen in this study is still more favorable than reported elsewhere in the literature.

**Table 4:** COD balance and theoretical H_2_ production of *C. termitidis* mono-cultured and co-cultured with *C. beijerinckii* on 2 g l^−1^ cellulose

		Metabolites^a^ (g COD l^−1^)	H_2_^b^ (g COD l^−1^)	Cellulose (g COD l^−1^)	Biomass^c^ (g COD l^−1^)	Total COD (g COD l^−1^)	COD balance^d^ (%)	Theoretical H_2_ (ml)			Experimental H_2_ (ml)	Difference (%)
								From acetic acid	From butyric acid	Total		
Mono	Initial	0.01	0	2.55	0.01	2.57	97	247	0	247	245	1
	Final	1.08	0.31	0.49	0.61	2.49						
Co	Initial	0.19	0	2.55	0.02	2.76	108	136	186	322	324	0
	Final	1.79	0.41	0.17	0.63	3						

^a^Metabolites COD accounts for the sum of acetate, butyrate, lactate, formate and ethanol as g COD l^−1^

^b^Calculated based on 8 g COD g^−1^ H_2_

^c^Biomass COD was calculated by multiplying dry weight (g l^−1^) × 0.9 × 1.42 (g COD g^−1^ biomass)

^d^COD mass balance = (Final TCOD/Initial TCOD) × 100 %

**Fig. 5:**
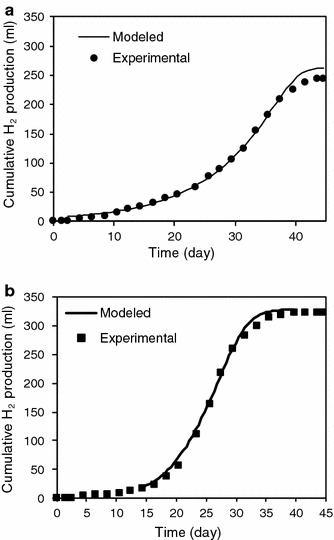
Experimental and modeled H_2_ profiles for **a** mono-culture and **b** co-culture

Based on the modeled acetate and butyrate profiles, modeled H_2_ profiles shown in Fig. 5 were calculated in a similar manner as the theoretical H_2_ shown in Table 4, with 848 ml H_2_ g^−1^ acetate and 578 ml hydrogen g^−1^ butyrate (from stoichiometry of Eqs. 2 and 3), and 1.067 g COD g^−1^ acetate and 1.82 g COD g^−1^ butyrate. The modeled H_2_ profiles closely match the experimental H_2_, as verified with the low APE values ranging from 10 to 15 % and RMSE values (9–13 ml).

### Microbial products and kinetics

Anaerobic lactate consumption has been reported by different inoculums, such as soil, kitchen waste compost, *Clostridium diolis* JPCC H-3, *Clostridium butyricum* JPCC H-1, *C. acetobutylicum* P262, and also *C. beijerinckii* JPCC H-4 (Diez-Gonzalez et al. 1995; Grause et al. 2012; Lee et al. 2010; Matsumoto and Nishimura 2007). Nevertheless, in some cases, acetate has been simultaneously consumed. The metabolic pathways reported in the literature are shown in Eqs. 4, 15 and 16 (Costello et al. 1991; Diez-Gonzalez et al. 1995; Grause et al. 2012; Matsumoto and Nishimura 2007; Thauer et al. 1977):

15 $$ CH_{3} CH\;\left( {OH} \right)COOH\; + \;0.5CH_{3} COOH\; \to \; 0.75CH_{3} CH_{2} CH_{2} COOH\; + \;0.5H_{2} \; + \;CO_{2} \; + \;0.5H_{2} O $$

16 $$ CH_{3} CH\left( {OH} \right)COOH\; + \;0.43CH_{3} COOH\; \to \; 0.7CH_{3} CH_{2} CH_{2} COOH\; + \;0.57H_{2} \; + \;CO_{2} \; + \;0.7H_{2} O $$

The evident lactate consumption in co-culture fermentations shown in Fig. 4b, could be assumed to follow Eq. 4 since acetate was produced simultaneously.

Apparently, co-culture fermentation exhibited acetate consumption after day 27 (Fig. 4b), which could be explained by Eqs. 15 and 16, although lactate was below the detection limit during this period of time. In contrast, mono-culture fermentation did not exhibit this phenomenon because *C. termitidis* does not produce butyrate; thus acetate consumption in co-culture fermentations could be attributed to the presence of *C. beijerinckii*. Interestingly, the co-culture experiment of *C. thermocellum* JN4 and *T. thermosaccharolyticum* GD17 on cellulose reported by Liu et al. (2008) also consumed lactate with acetate production whereas *C. thermocellum* JN4 in mono-culture did not; no explanation of this phenomenon was attempted by the authors.

Desvaux et al. (2000) found a µ_max_ of 0.056 h^−1^ with *C. cellulolyticum* grown on 2.4 g cellulose l^−1^ with a biomass yield of 36.5 g of cells mol^−1^ hexose equivalent (or 0.2 g cells g^−1^ hexose). Kinetics on cellulose have been also explained by alternative models to Monod. For example, Holwerda and Lynd (2013) found that the best fit to their results on *C. thermocellum* was with a substrate utilization rate that is both first order with respect to substrate and first order in cells. Recently, Gupta et al. (2015) found a µ_max_ of 0.05 d^−1^ on cellulose using mesophilic anaerobic digested sludge (ADS) and a Ks of 2.1 g l^−1^, which is four times lower than that achieved by *C. termitidis* in the present study.

This study is the first to model *C. termitidis* microbial kinetics on cellulose and in co-culture with *C. beijerinckii*. High H_2_ yields at mesophilic temperature directly from cellulose of 1.8 and 2.05 mol hydrogen mol^−1^ hexose equivalent_consumed_ in mono-culture and co-culture, respectively, were achieved as compared to the literature. Cellulose degradation by the co-culture was 15 % higher than the mono-culture of *C. termitidis*. The viability of *C. termitidis* and *C. beijerinckii* producing H_2_ together was evidenced.

## Additional file

**Additional file 1.** Additional figures and tables.
