# Supplementary material for: Hydrogen production and microbial kinetics of Clostridium termitidis in mono-culture and co-culture with Clostridium beijerinckii on cellulose
Source: AMB Express. 2017 Apr 20;7:84. doi: 10.1186/s13568-016-0256-2 (PMC5399015; doi:10.1186/s13568-016-0256-2)
Supplement: Supplementary file 1 — Additional file 1. Additional figures and tables. [file 13568_2016_256_MOESM1_ESM.pdf]

SUPPLEMENTARY MATERIAL

Hydrogen Production and Microbial Kinetics of *Clostridium termitidis* in Mono-culture and Co-culture with *Clostridium beijerinckii* on Cellulose

Applied Microbiology and Biotechnology Express

Maritza Gomez-Flores,[1] George Nakhla,[1,2] Hisham Hafez[2]

[1]Department of Chemical and Biochemical Engineering, Faculty of Engineering, University of Western Ontario, London, Ontario, N6A 5B9, Canada.

[2]Department of Civil and Environmental Engineering, Faculty of Engineering, University of Western Ontario, London, Ontario, N6A 5B9, Canada.

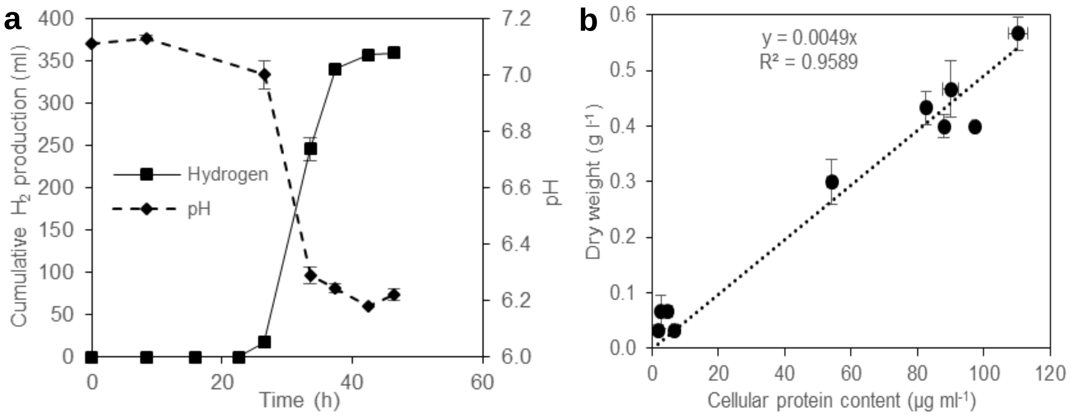

Figure S1 *C. beijerinckii* in 2 g l^{-1} glucose. **a** pH and cumulative H_2 production profiles. **b** Dry weight and cellular protein content correlation. Data points represent the mean values of duplicate experiments, lines above, below and to the sides represent the actual duplicates

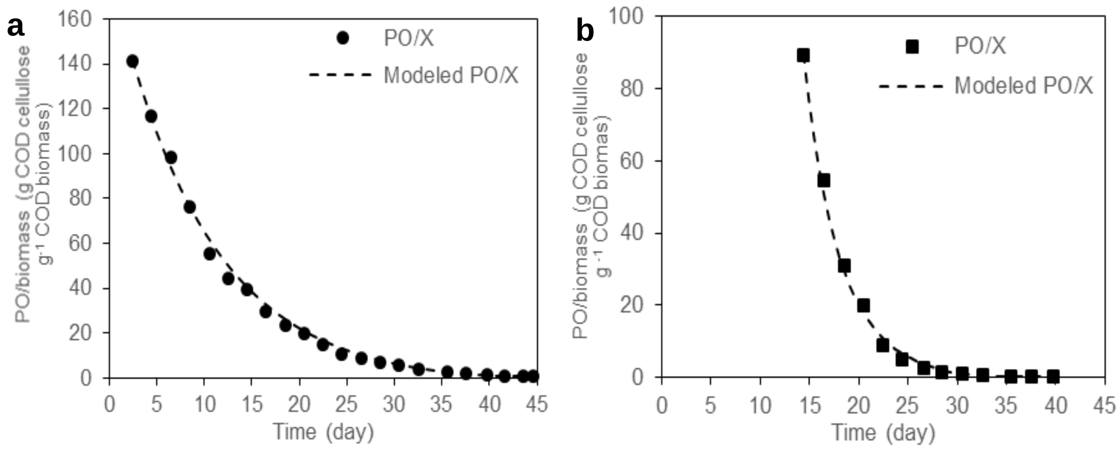

Figure S2 Experimental and modeled PO/biomass profiles. **a** Mono-culture. **b** Co-culture

Table S1 APE and RMSE for biomass, substrate and metabolites of *C. termitidis* mono-cultured on 2 g l^{-1} cellulose and co-cultured with *C. beijerinckii* on 2 g l^{-1} cellulose

		Mono-culture	Co-culture
APE (%)	Biomass	7	8
	Cellulose	4	5
	PO/X	9	11
	Lactic acid	81 (12)	23
	Formic acid	81 (11)	41
	Acetic acid	10	19
	Ethanol	19	24
	Butyric acid	NA	25
	H_2	15	10
RMSE (g COD l^{-1})	Dry weight	0.016	0.034
	Cellulose	0.062	0.088
	PO/X[a]	2.42	1.39
	Lactic acid	0.008	0.013
	Formic acid	0.011	0.017
	Acetic acid	0.042	0.069
	Ethanol	0.030	0.054
	Butyric acid	NA	0.115
	H_2 (ml)	9	13

APE: Average percentage error

RMSE: Root mean square error

NA: Not Applicable

[a] g COD cellulose g^{-1} biomass

( ): values excluding lag phases
